# Effects of Oral Vitamin D Supplement Therapy on Clinical Outcomes of Intravitreal Bevacizumab in Diabetic Macular Edema

**DOI:** 10.18502/jovr.v16i1.8249

**Published:** 2021-01-20

**Authors:** Saeed Karimi, Vahid Movafaghi, Amir Arabi, Toktam Shahraki, Sare Safi

**Affiliations:** ^1^Ophthalmic Research Center, Research Institute for Ophthalmology and Vision Science, Shahid Beheshti University of Medical Sciences, Tehran, Iran; ^2^Department of Ophthalmology, Torfeh Medical Center, Shahid Beheshti University of Medicine Sciences,Tehran, Iran; ^3^Ophthalmic Epidemiology Research Center, Research Institute for Ophthalmology and Vision Science, Shahid Beheshti University of Medical Sciences, Tehran, Iran

**Keywords:** 25-Hydroxyvitamin D, Insufficiency, Diabetic Macular Edema, Intravitreal Bevacizumab

## Abstract

**Purpose:**

To assess the effects of oral vitamin D supplement therapy on clinical outcomes of intravitreal bevacizumab (IVB) injections in patients with diabetic macular edema (DME).

**Methods:**

Seventy-one patients with center-involving DME received IVB injections three times monthly. Cases with serum 25-hydroxyvitamin D (25(OH)D) levels <30 ng/ml were divided into treatment and control groups. The treatment group received 50000 IU of oral vitamin D once a week for eight weeks. One month after the third IVB injection, changes in the best-corrected visual acuity (BCVA) and central macular thickness (CMT) were analyzed for each group.

**Results:**

Thirty-seven patients had sufficient levels of 25 (OH) D, while 34 patients had insufficient levels. Nineteen cases with deficient levels of 25(OH)D were treated with oral vitamin D, while 15 patients were assigned to the control group. The mean of serum 25(OH)D in patients was 27.9 ng/ml [mean 20.3 ± 5.4 and 17.3 ± 5.4 ng/ml in control and treatment groups, respectively (*P* = 0.231)]. After three IVB injections, BCVA improved significantly in each group, but the difference between the study groups was not statistically significant. CMT decreased significantly in all the groups. The mean CMT reduction was more prominent in the vitamin D-treated group, but the difference between groups did not reach statistical significance (*P* = 0.29).

**Conclusion:**

In DME patients with vitamin D deficiency, vitamin D supplement therapy had some beneficial effects on CMT reduction following three injections of IVB; nevertheless, these effects were not statistically significant. Definite conclusion needs further prospective studies with a larger sample size.

##  INTRODUCTION

Diabetic macular edema (DME) may develop in diabetic patients, independent of the severity of diabetic retinopathy (DR).^[1]^ DME is the main cause of decreased vision in diabetic patients.^[1]^ Elevated levels of various inflammatory and angiogenic factors lead to serious damage of retinal vascular endothelial cells, and the consequent impairment of blood–retinal barrier (BRB) causes fluid accumulation in the retinal tissue.^[2]^

Vitamin D is a well-known endocrine secosteroid which plays an essential role in many physiologic processes, including the control of cellular apoptosis and differentiation, as well as angiogenesis and metastasis potential of human cancer cells.^[3,4,5,6]^ Various cardiovascular, infectious, and autoimmune diseases have been revealed to be linked with vitamin D deficiency.^[7]^

Both the vitamin D activator enzyme (1-α-hydroxylase ) and its receptor have been found in the retina,^[8,9]^ suggesting that 25(OH)D abnormal levels may participate in the development and progression of various retinal disorders, including DR. Deficient serum 25(OH)D levels have been shown to be correlated with more advanced DR and its vision-threatening outcomes.^[10,11]^

In this study, we measured the serum vitamin D levels in patients scheduled to receive intravitreal bevacizumab (IVB) for DME. We investigated the influence of oral vitamin D supplement therapy on the outcomes of IVB injections in these patients.

##  METHODS

The current prospective comparative case series study was carried out between March 2017 and August 2018. The protocol was approved by the Ethics Committee of the Ophthalmic Research Center at the Shahid Beheshti University of Medical Sciences and followed the Declaration of Helsinki. A written consent was obtained from all patients.

One eye from each patient was enrolled in the study. A diagnosis of center-involving DME was made if the central macular thickness (CMT) (within central 1-mm of macula) was >300 μm on optical coherence tomography (OCT) image (Spectralis OCT; Heidelberg Engineering, Vista, CA). Subjects were eligible for enrolment if BCVA was between 20/40 and 20/320 according to the Snellen chart in the eye enrolled in the study. The exclusion criteria were history of intravitreal anti-VEGF injections in the last three months of enrolment, history of intraocular surgery other than uncomplicated cataract surgery, patients with proliferative DR, retinal vascular occlusions, glaucoma, a creatinine (Cr) level > 3 mg/dl, thyroid and parathyroid diseases, liver disease or any other problem of vitamin D absorption, recent use of supplements containing vitamin D or 25(OH)D, use of medications with known effect on serum 25(OH)D levels such as anticonvulsants and corticosteroids, and serum 25(OH)D level ≤ 10 ng/ml.

All patients were scheduled to receive IVB (AvastinⓇ, Genentech/Roche, CA, USA) three times monthly. All subjects underwent intravitreal injections at the Torfeh Eye Hospital. Ophthalmologists who performed the injections were masked to the groups. The study was performed during a single season to avoid variations in serum vitamin D levels due to seasonal exposures.

Before enrolment, all patients underwent complete ophthalmic examination. Parameters including age, sex, BCVA, and CMT were measured for each subject. On the day of first injection, venous blood specimen was analyzed for 25(OH)D, Cr, and HbA1c levels. Patients with >30 ng/ml of 25-hydroxyvitamin D (25(OH)D) were considered as vitamin D-sufficient group. Patients with <30 ng/ml were enrolled in the control group. The subjects were assigned to treatment groups on a random basis without considering the 25(OH)D levels. Thus, we had three study groups: group 1 (vitamin D-sufficient group with serum vitamin D ≥ 30 ng/ml), group 2 (vitamin D-deficiency group treated with oral vitamin D supplement), and group 3 (vitamin D-deficiency control group). The treatment group received a pearl of vitamin D3 (D-Vigel 50000 IU, Daana Pharmaceutical Company, Iran) once a week for eight consecutive weeks during the first two months of the IVB treatment period. Fundus examination and OCT imaging were repeated before any procedure.

Visual acuity measurements were obtained through Snellen chart examination by a trained optometrist who was masked as to which group the patients were assigned to, and were converted to LogMAR values. Severity of DR was determined by a single ophthalmologist using three field fundus photographs (optic disc centered, fovea centered, and centered on temporal edge of the macula), and was categorized according to the International DR Severity Scale.^[12]^ Ophthalmic evaluations were repeated one month after the third intravitreal injection. The mean changes in BCVA and CMT from baseline to one month after the third injection were measured as primary and secondary outcomes, respectively. After the completion of the study protocol, patients of the control group were also treated with oral vitamin D supplement.

**Figure 1:**
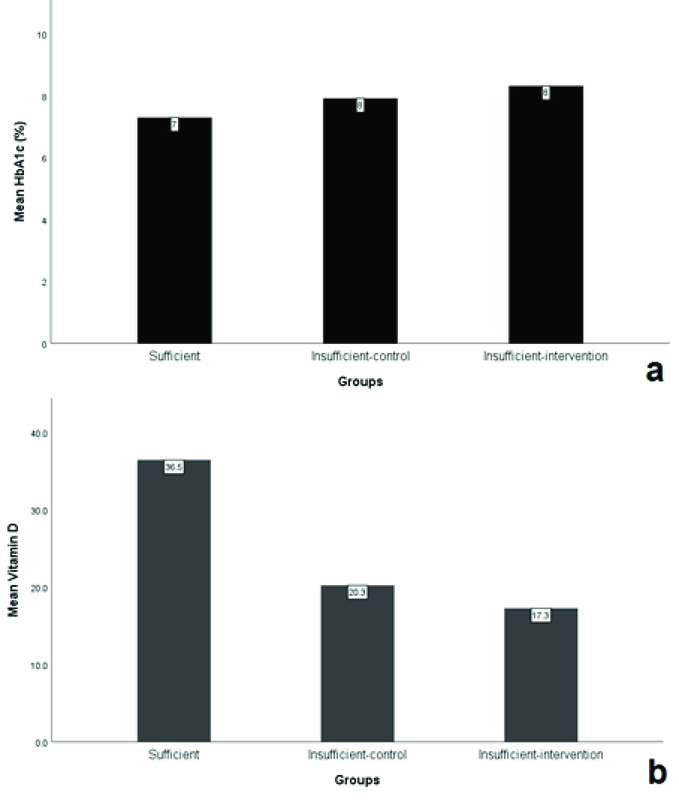
The mean HbA1c (a) and vitamin D levels (b) in different study groups.

To present data, mean and standard deviation were used. *T*-test was used for comparing serum vitamin D and HbA1c between the groups, and the correlation between HbA1c and vitamin D levels was evaluated by linear regression analysis. To evaluate the role of treatment on LogMAR and CMT changes, paired *t*-test analysis was used. The differences were considered as significant if *p*-value was < 0.05 (Figure 1). Finally, to determine the adequacy of the sample size and the power of the study, a post-hoc analysis was performed.

**Figure 2:**
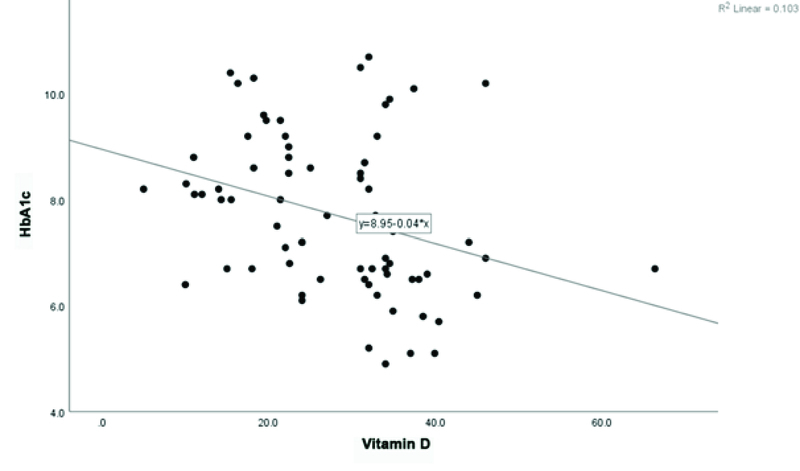
The correlation chart of vitamin D and HbA1c levels.

**Figure 3:**
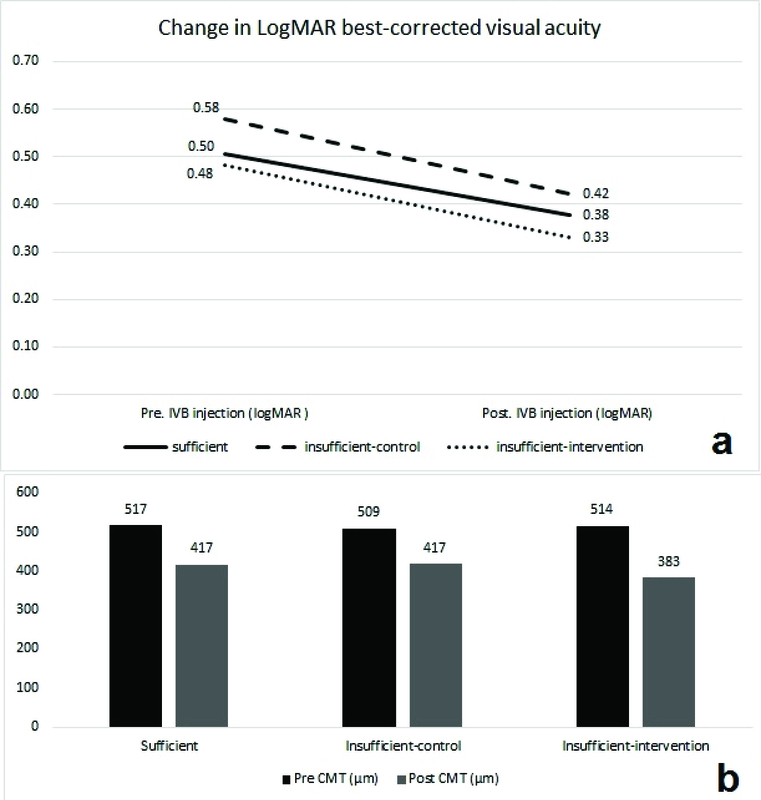
Changes in visual acuity (a) and central retinal thickness (b) following IVB therapy. CMT, central macular thickness; LogMAR, logarithm of minimal angel resolution; IVB, intravitreal bevacizumab

##  RESULTS

Eighty-three patients participated in the study. Four patients were excluded due to urgent need for supplement therapy (vitamin D level < 10 ng/ml). Eight patients (one patient from treatment group, five patients from control group, and two patients from sufficient group) did not complete the study. Out of the 71 subjects analyzed at the end of study, 37 patients had sufficient levels of 25(OH)D, 19 had insufficient 25(OH)D and were treated with oral vitamin D supplement (treatment group), and 15 cases with insufficient 25(OH)D levels were enrolled as the control group. Demographic characteristics and baseline parameters are summarized in Table 1. The study groups were matched in terms of age, sex, and severity of DR (Table 1).

**Table 1:** Demographic and clinical features


**Factors**	**Levels**	**Total**	**Groups**				**** ***P*** **-value**
		**Sufficient**	**Insufficient-control**	**Insufficient-treatment**	**Insufficient-control and Insufficient-treatment**	
**Age**	Mean ± SD	63 ± 8	65 ± 6	59 ± 8	64 ± 9	62 ± 9	0.076
	Median (range)	64 (40,84)	64 (52,84)	59 (45,74)	64 (40,84)	63 (40,84)	
**Sex**	Male	37 (52.1%)	18 (48.6%)	7 (46.7%)	12 (63.2%)	19 (55.9%)	0.518
	Female	34 (47.9%)	19 (51.4%)	8 (53.3%)	7 (36.8%)	15 (44.1%)	
**Eye**	OD	37 (52.1%)	22 (59.5%)	5 (33.3%)	10 (52.6%)	15 (44.1%)	0.252
	OS	34 (47.9%)	15 (40.5%)	10 (66.7%)	9 (47.4%)	19 (55.9%)	
**DR**	NPDR	32 (45.1%)	19 (51.4%)	5 (33.3%)	8 (42.1%)	13 (38.2%)	0.511
	PDR	39 (54.9%)	18 (48.6%)	10 (66.7%)	11 (57.9%)	21 (61.8%)	
**HbA1c**	Mean ± SD	7.7 ± 1.5	7.3 ± 1.6	7.9 ± 1.3	8.3 ± 1.1	8.2 ± 1.2	0.043
	Median (range)	7.6 (4.9,10.7)	6.7 (4.9,10.7)	8.2 (6.1,9.6)	8.1 (6.7,10.4)	8.1 (6.1,10.4)	
**Vitamin D (µg)**	Mean ± SD	27.9 ± 10.8	36.5 ± 6.7	20.3 ± 5	17.3 ± 5.4	18.6 ± 5.4	<0.001
	Median (range)	31 (5,66.3)	34.2 (31,66.3)	22 (10,26.2)	18 (5,27)	19.5 (5,27)	
DR, diabetic retinopathy; NPDR, nonproliferative diabetic retinopathy; OD, right eye; OS, left eye; PDR, proliferative diabetic retinopathy; HbA1c, hemoglobin A1c; SD, standard deviation							

**Table 2:** Best-corrected visual acuity changes after three intravitreal bevacizumab injections


**BCVA (LogMAR)**	**Total**	**Groups**			**** ***P*** **-value**
	**Sufficient**	**Insufficient-control**	**Insufficient-treatment**	
**Baseline **	0.51 ± 0.28	0.5 ± 0.28	0.58 ± 0.25	0.48 ± 0.32	0.587
**Final F/U**	0.37 ± 0.24	0.38 ± 0.24	0.42 ± 0.24	0.33 ± 0.23	0.554
**Change**	–0.14 ± 0.13	–0.13 ± 0.12	–0.16 ± 0.17	–0.15 ± 0.11	0.659
**** ***P*** **-value**	<0.001	0.002	<0.001	
CMT (µm)					
**Baseline **	514 ± 102	517 ± 112	509 ± 74	514 ± 105	0.97
**Final F/U **	408 ± 96	417 ± 111	417 ± 61	383 ± 87	0.42
**Change **	–106± 83	–100± 97	–91± 53	–131± 68	0.29
**** ***P*** **-value**	<0.001	<0.001	<0.001	
BCVA, best-corrected visual acuity; F/U, follow-up; CMT, central macular thickness					

The average HbA1c levels in the sufficient (*n* = 37) and the insufficient (*n* = 34) groups were 7.3% and 8.2%, respectively (*P* < 0.05, Figure 1a). The difference of HbA1c levels between the treatment and the control groups was not statistically significant (*P* > 0.05, 95% CI).

The mean serum 25(OH)D level was 27.9 ng/ml and it did not show any statistical correlation with patients' sex (*P* = 0.653). Regression analysis showed that serum 25(OH)D levels had negative correlation with the HbA1c levels (Pearson's correlation coefficient = –0.032) in all the patients. The *P*-value for the correlation was 0.007, showing a significant relationship (Figure 2).

The mean levels of serum 25(OH)D were 36.5 ± 6.7, 17.3 ± 5.4, and 20.3 ± 5.4 ng/ml in the sufficient group, the insufficient treatment group, and the insufficient control group, respectively (Figure 1b). The mean 25(OH)D level was significantly higher in the sufficient group, while the difference between the control and the treatment groups was not statistically significant (*P* = 0.231, 95% CI) (Table 1).

The mean BCVA values at the baseline were 0.51 ± 0.28, 0.48 ± 0.32, and 0.58 ± 0.25 LogMAR in the sufficient group, the insufficient treatment group, and the insufficient control group, respectively (*P* > 0.05, 95% CI). One month after the third IVB injection, BCVA improved significantly in all the study groups. The mean changes in BCVA were –0.13 ± 0.12, –0.15 ± 0.11, and –0.16 ± 0.17 LogMAR in the sufficient group, the insufficient treatment group, and the insufficient control group, respectively (*P* = 0.66). The mean changes in BCVA were not significantly different between the study groups (Table 2 and Figure 3a).

The mean CMT values were 517 ± 112 µm, 514 ± 105 µm, and 509 ±74 µm in the sufficient group, the insufficient treatment group, and the insufficient control group, respectively (*P* = 0.97, 95% CI). One month after the third IVB injection, the mean CMT decreased significantly in all the study groups. The mean CMT changes were –100 ± 97, –131 ± 67, and –91 ± 53 µm in the sufficient group, the insufficient treatment group, and the insufficient control group, respectively (*P* = 0.29). Although the mean CMT decreased more in patients who received oral vitamin D supplement (–131 µm vs –100 µm and –91 µm), the difference was not statistically significant (*P* = 0.29, Table 2 and Figure 3b).

Post-hoc analysis showed that the sample size should be 41 in each group to find a significant difference (50 microns) in the changes of CMT to achieve the study power of 95%. However, the present study had a power of 60%. Endophthalmitis or significant ocular or systemic complications were not observed.

##  DISCUSSION

We observed that vitamin D supplement therapy in the subset of diabetic patients with DME and insufficient levels of vitamin D could not improve the outcome of IVB therapy. According to our knowledge, this study was the first to investigate the effect of oral vitamin D supplement therapy on clinical outcomes of IVB injection in DME.

There is still a degree of uncertainty about the influence of serum vitamin D in occurrence, progression, and prognosis of DR. The controversy begins from the concept that serum vitamin D deficiency may not be correlated with a concurrent ocular deprivation. It is believed that the eye can produce vitamin D through exposure to ultraviolet light, and the BRB can limit the transmission of vitamin D from blood to the eyes.^[13,14]^ Accordingly, some authors have suggested that intraocular 25(OH)D levels may not depend on systemic 25(OH)D levels.^[1]^

It has also been reported that the 1,25-dihydroxycholecalciferol can upregulate the expression of the VEGFs; it has been shown that the regulation of VEGF promoter by vitamin D receptor increases the secretion of VEGFs in vascular smooth muscles.^[15,16]^ A similar effect has not been established in retinal cells, however, this finding can hypothesize a correlation between high ocular vitamin D levels and high concentration of ocular VEGFs.^[1]^

Previous studies have revealed paradoxical results about the relationship between the severity of DR and serum 25(OH)D levels. Some authors have reported an inverse relationship,^[10,11][17][18][19]^ while others have not shown such a correlation.^[20,21]^ A meta-analysis on the topic reported that those patients with DM type 2 and vitamin D deficiency have a higher risk of DR development compared to subjects with adequate levels of the vitamin.^[22]^ On the other hand, a recent study performed by Kim et al reported that patients with DME had a greater aqueous humor amounts of vitamin D than the patients without DME.^[1]^ It should also be considered that studies on vitamin D levels encounter some challenges such as different cultural backgrounds as well as different clothing and diet styles. Vitamin D in the body can be supplied both from dietary sources and from synthesis in the skin. Accordingly, it is difficult to control the dietary, environmental, seasonal, and cultural factors, in addition to predict serum vitamin D levels through single measurement, which can cause inconclusive results.

Since the prior studies have not resulted in an exact conclusion about vitamin D and its role in DR, we investigated the treatment of DME with an anti-VEGF agent, bevacizumab, in the presence of vitamin D deficiency. We also tested the role of concurrent vitamin D3 therapy in optimizing the IVB therapy for DME. We found that the concurrent vitamin D supplement therapy in this subset of patients did not significantly improve the outcomes of IVB in DME cases with vitamin D deficiency. Although improvement in BCVA and decrease in CMT was more prominent in the treatment group, the difference between the control and the treatment groups was not statistically significant. Future studies on larger group of patients may reveal an association between vitamin D supplementation and improved outcomes of IVB injections in DME patients.

We found a negative correlation between HbA1c levels and serum 25(OH)D, implying that patients with 25(OH)D deficiency had a higher rate of uncontrolled hyperglycemia. This finding may be in accordance with prior reports regarding the correlation of vitamin D deficiency with poor glycemic control and DR severity.^[22]^ It has been postulated that vitamin D may improve insulin secretion, stimulate insulin receptor, and improve glucose uptake in type 2 diabetes.^[9,23]^ According to these assumptions, vitamin D may improve insulin resistance. However, it should be proven in experimental studies.

A small sample size in addition to the lack of a control for those habits and restrictions which may affect vitamin D storage in the body are the main limitations of the present study. Although vitamin D deficiency was treated according to the standard protocol, effectiveness of vitamin D supplement therapy was not assessed at the end of the study. Short-term follow-up could also be considered as another limitation of the present study; however, longer follow-up was not possible due to the ethical issues.

In conclusion, we observed a negative correlation between HbA1c and 25(OH)D levels. Although vitamin D supplement therapy, added to IVB therapy, had some beneficial effects in terms of CMT reduction in DME patients with 25(OH)D deficiency, we could not find any statistically significant effect of the adjunctive therapy on the functional and anatomical outcomes of these patients. Further studies are required to investigate the effect of D3 supplement therapy on optimizing the treatment of patients with DR.

##  Financial Support and Sponsorship 

None.

##  Conflicts of Interest

The authors have no conflicts of interest to declare.
